# Visitors to Hospital Patients

**Published:** 1907-01-26

**Authors:** W. H. Sands

**Affiliations:** Secretary. Whitehaven and West Cumberland Infirmary, Whitehaven


### Editor's Letter-Box.

[Our Correspondents are reminded that prolixity is a great bar to publication, and that brevity of style and conciseness of statement greatly facilitate early insertion.]

### VISITORS TO HOSPITAL PATIENTS.

*To the Editor of* The Hospital.

Sir,—A reply to the following, through your paper, will greatly oblige :—

"Is it usual and desirable for hospitals to permit more than two visitors on visiting days, and in addition special visits from friends when circumstances seem to justify it? Can you refer me to any information on the subject?

Yours truly,
W. H. Sands,
Secretary.

Whitehaven and West Cumberland Infirmary,
Whitehaven, January 18, 1907.

.·. [The regulations as to visiting days and visitors are given in the particulars of each institution published in Burdett's "Hospitals and Charities." The general rule is to have fixed visiting days in each week, of which Sunday is usually one, say, between the hours of two and four. The rules further provide for special visits to patients seriously ill, at the discretion of the medical officers. The number of visitors is restricted to two to each patient, but several friends may see a patient on a given day provided not more than two are ever present in the ward at the same time.—Ed. The Hospital.]

